# A comparative analysis of heartworm medication use patterns for dogs that also receive ectoparasiticides

**DOI:** 10.1186/s13071-018-3076-1

**Published:** 2018-08-31

**Authors:** Robert Lavan, Kathleen Heaney, Srinivasan Rajagopalan Vaduvoor, Kaan Tunceli

**Affiliations:** 1 Outcomes Research, Animal Health, Center for Observational and Real-World Evidence, Merck & Co., Inc., Kenilworth, NJ 07033 USA; 2 MSD Animal Health, 2 Giralda Farms, Madison, NJ 07940 USA; 3 Med Data Analytics, Inc., 2 Blue Diamond Hill Court, Milltown, NJ 08850 USA

**Keywords:** Acaricide, Dog, Flea, Tick, Heartworm, Adherence

## Abstract

**Background:**

Heartworm medications and many oral or topical flea and tick products are provided as monthly doses while a newer oral flea/tick product, fluralaner (BRAVECTO® Chew), is re-dosed at a 12-week interval. This study focused on whether there was a difference in the number of heartworm medication doses that were purchased in the 12-months follow-up period for dogs that receive either fluralaner or other flea/tick medications that are dosed monthly.

**Methods:**

Clinic transaction records of heartworm medication purchases for over 200,000 dogs were examined to compare the purchase of heartworm preventative protection by dog owners that also receive flea and tick medications of differing efficacy durations.

**Results:**

Annual purchases of heartworm medication for dogs by owners that receive a flea and tick medication dosed at 12-week intervals was incrementally higher than the number of doses purchased for dogs receiving monthly flea and tick medications. The average number of monthly doses per year was slightly over 7 months for both categories of product. The distribution of purchases of monthly doses was also similar between groups.

**Conclusions:**

Dog owners who purchase a longer-acting flea and tick medication purchase as much heartworm medication annually for their dogs as dog owners who purchase monthly flea and tick medication. On average, dog owners who gave their dog fluralaner obtained significantly more months of heartworm preventative protection compared with dog owners who gave their dog a monthly flea and tick medication, although the biological significance of this increase in doses is very small.

## Background

In the USA and in many other parts of the world, dogs are at risk for both ectoparasites, like fleas and ticks, as well as endoparasites, like heartworm. Medications to prevent these infestations and infections exist which can be given by the veterinarian or pet owner at home. Most of these products are dosed on a monthly basis; however, some are given less frequently because they confer a longer duration of efficacy. A 2016 U.S American Pet Products Association Survey estimated that 42% of dog owners in the USA currently give heartworm medication to their dog [1]. It is not known if dog owners would have trouble providing monthly heartworm doses which adhere to the veterinary recommendation when they are also giving flea and/or tick medication which is not dosed on a monthly basis. These products all treat flea infestations and most also treat tick infestations on dogs; therefore, the products are referred to throughout the manuscript as flea/tick products.

An isoxazoline with an extended retreatment interval, fluralaner (BRAVECTO® Chew, Merck Animal Health, Giralda Farms, NJ, USA) was introduced for dogs in the USA in June, 2014. Fluralaner kills adult fleas and is indicated for the treatment and prevention of flea infestations (*Ctenocephalides felis*) and the treatment and control of tick infestations [*Ixodes scapularis* (black-legged tick), *Dermacentor variabilis* (American dog tick), and *Rhipicephalus sanguineus* (brown dog tick)] for 12 weeks in dogs and puppies 6 months of age and older, and weighing 4.4 pounds or greater. Fluralaner is also indicated for the treatment and control of *Amblyomma americanum* (lone star tick) infestations for 8 weeks in dogs that are 6 months of age and older, and weighing 4.4 pounds or greater [2].

In terms of flea/tick medication, fluralaner is unique in that each dose lasts up to 12 weeks, which is almost three times the re-dosing interval of most other flea/tick products which are dosed monthly. The benefits of extended duration flea and tick protection have been reported [3, 4], with the top three being reported by dog owners as “convenience”, “12-week dosing” and “dosing less often”. A question remains as to whether the use of an extended duration flea and tick product would impact the pet owner’s ability to administer monthly heartworm prophylaxis compared to dog owners who use monthly flea and tick products.

Owner self-reported data on adherence to veterinary recommendations is fraught with biases and can be unreliable. However, transactional data is an objective measure that can be used to assess pet owner adherence to the veterinary recommendation. An earlier study reported that 96% of surveyed veterinarians in the USA recommended 12 months of protection against fleas and ticks [4]. Adherent dog owners try to administer flea and tick medications for as many months as possible in a given year. While it is very difficult to measure doses given to dogs, we can use a history of purchases to estimate adherence by assuming that purchased doses were given.

The study goals were to utilize transactional data to (i) describe the treatment patterns for heartworm medications prescribed for dogs in the USA and (ii) assess the variation in the number of doses and the duration of coverage for heartworm medications between dog owners using an extended duration flea/tick prevention medication (i.e. fluralaner) and dog owners using other flea/tick prevention medications with a monthly re-dosing schedule.

## Methods

The raw, blinded transactional data for heartworm medication purchases were obtained from clinic invoices from a research panel provided by Vet Informatics [5]. Collected data did not contain any proper names or addresses for dog owners or their pets. Code numbers concealed owner identity while allowing serial transactions to be matched to an individual dog through the study period. The study period extended from June 2014, the first month that fluralaner was sold in the USA, through the end of November, 2017. All of the data analysis was performed by the Merck Animal Health Center for Observational and Real-World Evidence (CORE).

The clinic transactional data came from approximately 650 veterinary clinics, segmented by zip codes, in the southern USA (74.9%) and from clinics in the Midwest (16.9%), West (4.0%), Northeast (3.6%), and U.S. Protectorates (0.09%). Approximately 0.5% of all transactional data had no zip code listed. Heartworm medication doses were assessed as the mean yearly purchase volume, as well as the frequency of doses acquired. The heartworm medication purchases for each dog are represented by a single index date (first date that heartworm medication was purchased) and a single 12 month follow-up period (12 months following the index date). Dogs whose index period falls early in the study (2014 or early 2015) may have had the opportunity to count additional 12 month periods of heartworm purchases (additional index periods) but these were not assessed. Each dog contributed one 12 month follow-up period.

Dog owners were identified as “pure users” for particular flea/tick medications, meaning that they had purchased at least one dose of a flea/tick medication without switching to another product during a 12 month period. The focus flea/tick medications included BRAVECTO® Chew, Comfortis® (spinosad; Elanco), Frontline® Plus (fipronil and (s)-methoprene; Boehringer Ingelheim), Nexgard® (afoxolaner; Boehringer Ingelheim) and Simparica® (sarolaner; Zoetis). Other than fluralaner, the other flea/tick products all have a monthly re-dosing schedule. For the purposes of this study, the brands of monthly flea/tick medications were combined into a single comparator group. Dog owners purchased one or more of the heartworm prophylaxis products as follows: Advantage Multi®; Heartgard Chewables®; Heartgard Plus Chew®; Heartgard Tablets®; Interceptor Flavor Tabs®; Interceptor Plus®; Iverhart Max®; Iverhart Plus®; Iverhart® Unspecified; ProHeart 6®; Revolution®; Sentinel®; Tri-Heart Plus Chew® and Trifexis®.

The number of doses and treatment duration for pooled heartworm medications for dogs were compared using two flea/tick prevention treatment groups (fluralaner with 12 weeks re-dosing schedule *vs* other flea /tick medications with a monthly re-dosing schedule). The study counted doses of the heartworm products purchased during the 12-month follow-up period. Parasiticides which were excluded from this list included products solely labeled for cats, products indicated for the treatment of adult heartworm infestation, or anthelmintics which focus on non-heartworm species.

Inclusion and exclusion criteria were applied to the raw data to develop the final analytical database. These criteria removed records of non-canine species, duplicate entries and ensured proper dose counts, especially with multi dose packs. An additional filter was applied to the initial database to ensure that each patient record represented transactions for one dog and not multiple dogs. To eliminate situations where a transaction might have been made for more than one dog, canine patients were removed if more than 12 doses were purchased in a single transaction or more than 24 doses were obtained in 12 months. This is based on the assumption that a pet owner might acquire a maximum of 12 months of product for the first year and then get a second 12 months for the following year before the current 12 month period ended. Dog owners with recorded transactions in excess of this which were in the transaction record of a single dog were considered to be acquiring doses for multiple dogs. Dogs older than 6 months were allowed in the analysis. The Index Date had to be prior to November 1, 2016, in order for the pet owner to have a full 12 month window to acquire additional doses. All transactions of heartworm prevention medication were counted for the 12 months following the index date.

The clinic transaction records were aligned by patient ID number and placed in chronological order for each dog. Heartworm doses that were purchased in the 12 months that followed were recorded. The total doses purchased were then adjusted to produce a dose count of heartworm medication that could actually be used in the 12 month period. This meant that doses obtained late in the 12 month follow-up period might count for a fraction of the last month. For example, if the first monthly dose was purchased on January 1 and the last dose was purchased the following December 15, the pet would be credited with full doses up to the last dose, while the last dose would count for 15 of 31 days in the month or approximately half of the last month. Doses or proportion of doses that might have provided heartworm protection after the 12 month period were not included in calculating duration of heartworm protection. It was assumed that all doses were given on time and consecutively if multiple doses were obtained in one transaction.

Descriptive statistics were created for the total quantity of heartworm medication purchased, months of heartworm coverage, dog age and dog body weight. Distributions of months of heartworm protection purchases among fluralaner users and users of all monthly flea control products combined were created. A general linear model (GLM) was used to derive and compare the least squares means of the months of coverage of heartworm product purchases among fluralaner users and users of all monthly flea control products combined.

All analyses were carried out using SAS® 9.4 (SAS Institute, Cary, NC). Statistical significance was set at *P* < 0.05.

## Results

Approximately 961,000 dogs identified as “pure users” of a particular flea/tick medication were included in the raw data. After applying inclusion and exclusion criteria, the final analysis was performed on 202,550 dogs (fluralaner group = 56,756 dogs; four monthly medications = 145,794 dogs).

Limited canine patient demographic data was included with the transaction database. The average age of study dogs was 67.7 months (about 5.6 years) and the average weight was 18.0 kg. The age and weight of dogs in the two groups were similar (Table 1), with a difference of only 0.9 kg average body weight (about 5%) and 3 months (about 4%) in average age between the comparator groups. The average age and body weight for the two groups were significantly different (body weight *t*_(1)_ = -14.54, *P* < 0.0001; canine age *t*_(1)_ = 12.84, *P* < 0.0001 ) but considered clinically irrelevant and secondary to the large number of dogs in each treatment group.

**Table 1:** Mean age, body weight and months of heartworm preventive purchased across comparator groups

	Least squares mean (95% CI)		Difference (95% CI)
	Fluralaner (*n* = 56,756)	Four monthly flea/tick products (*n* = 145,794)	
Index age (months)	65.79 (65.43–66.14)^a^	68.52 (68.30– 68.74)^b^	2.74 (2.32–3.15)
Body weight (kg)	18.64 (18.53–18.75)^a^	17.69 (17.63–17.76)^b^	-0.95 (-1.08– -0.82)
Months purchased	7.35 (7.32–7.39)^a^	7.13 (7.11–7.15)^b^	-0.22 (-0.260– -0.182)

Statistical comparison of results for index age (*t*_(1)_ = 12.84, *P* < 0.0001); body weight (*t*_(1)_ = -14.54, *P* < 0.0001) and months of purchased heartworm preventive (*t*_(1)_ = -11.02, *P* < 0.0001) Different superscripts indicate significant differences at *P* > 0.05

The average annual duration for heartworm medication use for dogs in this study is presented in Table 1. Dogs that received fluralaner or monthly flea/tick products all had over 7 months of heartworm medication use; mean: 7.35 (95% CI: 7.32–7.39) and 7.13 (95% CI: 7.11–7.15) months, respectively. Again, because of the large number of dogs in this study, a mean difference of 0.22 months per year between these two groups was statistically significant (*t*_(1)_ = -11.02, *P* < 0.0001) but not clinically relevant. Essentially, dogs that receive Bravecto Chew (fluralaner) had, on average, as many months of annual heartworm medication coverage as dogs that receive monthly flea/tick products.

The percent distribution of annual heartworm medication coverage in months is presented in Table 2. About 10–12% of dogs that receive fluralaner or the monthly flea/tick products had only one dose of heartworm medication acquired for them (i.e. one month coverage). The ranges for proportion of dogs with 6 months coverage and 12 months coverage were 20.2–24.7% and 31.9–32.8%, respectively and very similar between groups. All paired comparisons between treatment groups were always significantly different (6 months, *χ*^2^ = 496, *df* = 1, *P* < 0.0001; 12 months, *χ*^2^= 14, *df* = 1, *P* = 0.0002). , even though the differences were small and clinically irrelevant.

**Table 2:** The percentage of dogs with annual heartworm medication coverage in months by flea/tick medication treatment category

Months of heartworm coverage	Fluralaner	Four monthly flea/tick products
1.0–1.9	10.3	12.6
2.0–2.9	8.3	10.3
3.0–3.9	4.4	4.9
4.0–4.9	2.1	2.7
5.0–5.9	1.4	1.7
6.0–6.9	24.7^a^	20.2^b^
7.0–7.9	4.3	3.8
8.0–8.9	3.5	3.3
9.0–9.9	4.2	3.6
10.0–10.9	4.9	4.2
11.0–12.0	31.9^a^	32.8^b^

Statistical comparison of results at 6 months (*χ*^2^ = 496, *df* = 1, *P* < 0.0001) and 12 months (*χ*^2^ = 14, *df* = 1, *P* = 0.0002). Different superscripts indicate significant differences at *P*<0.05.

## Discussion

Results from the study show that dog owners using the extended 12-week flea and tick protection of fluralaner acquired statistically significantly greater average annual coverage for heartworm prevention medication than dog owners who were using monthly flea/tick medications. However, the biological significance of the additional heartworm protection doses obtained by these owners is limited because the large number of records analyzed led to a declaration of statistical significance based on a slight increase in protection duration. The distribution of months of coverage for heartworm medications were also similar between the two groups.

The examination of clinic transaction records as a way to investigate pet owner medication use has an important limitation. The purchase history of a medication is an imperfect substitute for the number of doses of medication that a pet actually gets and is a reflection of the maximum doses that a pet might actually receive. Despite this limitation, examination and analysis of transactions provide an objective comparative approach to the measurement of owner compliance assuming that differences between purchased amounts and utilized doses are similar between treatment groups.

Dog owners who acquired fluralaner for their dogs had similar patterns of adherence to veterinary recommendations on heartworm prophylaxis as dog owners who used monthly flea/tick products. Using a flea/tick medication that is dosed at 12-week intervals does not seem to negatively impact dog owners who also use heartworm medication dosed at a different time interval. Purchase transactions of heartworm medications suggest that dogs that are given fluralaner for flea/tick control have at least as much heartworm prophylaxis purchased for them as dogs on shorter acting flea/tick medications.

## Conclusion

The use of a longer-acting flea and tick product by dog owners may lead to a slight increase in purchased heartworm protection but this increase is not likely to be biologically significant. On average, dog owners in this study who purchase canine flea and tick medication also purchased over 7 months of heartworm protection per year. The proportion of dog owners who bought one monthly dose to twelve monthly doses of heartworm medication in a year were also similar.

## Abbreviations

CORE: MSD Center for Observational and Real-World Evidence
